# Reshaping the definition of high-risk disease in the immune effector therapy era in multiple myeloma

**DOI:** 10.3389/fonc.2026.1956522

**Published:** 2026-10-01

**Authors:** Yoshito Nishimura, Gunjan Gaur, Francesca Cottini, Saurabh Zanwar

**Affiliations:** 1 Division of Hematology, Department of Medicine, Mayo Clinic, Rochester, MN, United States; 2 Division of Hematology, Department of Internal Medicine, The Ohio State University Wexner Medical Center, James Cancer Hospital and Solove Research Institute, Columbus, OH, United States

**Keywords:** bispecific antibody, CAR-T, high-risk myeloma, multiple myeloma, plasma cell leukemia

## Abstract

Multiple myeloma (MM) outcomes have improved with proteasome inhibitors, immunomodulatory drugs, anti-CD38 antibodies, and transplantation, yet a subset of patients with high-risk MM (HRMM) continues to relapse early and die prematurely. Immune effector therapies (IET)—B-cell maturation antigen (BCMA)- and G protein–coupled receptor class C group 5 member D (GPRC5D)-directed chimeric antigen receptor T cells (CAR-T) and bispecific antibodies—have transformed the treatment of relapsed/refractory MM. However, their benefit in HRMM remains incompletely defined. This review describes how HRMM is defined in the IET-era distinguishing baseline biological/genomic risk, clinical high-risk phenotypes, and IET-specific adverse predictors. The International Myeloma Society/International Myeloma Working Group consensus integrates adverse cytogenetics and beta-2 microglobulin, whereas functional high-risk disease, extramedullary disease (EMD), plasma cell leukemia (PCL), high tumor burden, inflammation, and prior BCMA exposure identify aggressive disease in relapsed patients. Across pivotal trials, CAR-T therapy, particularly ciltacabtagene autoleucel, and selected bispecific antibodies retain activity in high-risk cytogenetic subgroups, whereas EMD and PCL remain associated with inferior responses and short progression-free survival. Emerging dual-antigen and trispecific engagers, along with novel targets such as FcRH5 and CD70, aim to overcome antigen escape. Prospective, biology-informed studies are needed to achieve durable disease control in HRMM.

## Introduction

The treatment landscape of multiple myeloma (MM) has evolved with the incorporation of proteasome inhibitors (PI), immunomodulatory drugs (IMiDs), anti-CD38 antibodies, autologous transplantation, and maintenance therapy for high-risk patients. However, outcomes remain suboptimal in high-risk multiple myeloma (HRMM), including patients with high-risk cytogenetics, extramedullary disease (EMD), circulating plasma cells, plasma cell leukemia (PCL), high tumor burden, or early relapse, despite quadruplet induction regimens. While an initial response has become more common with modern induction regimens, securing long-term durable disease control has been challenging in this particular cohort (1–6).

Over the past several years, immune effector therapies (IET) have emerged and improved response depth, particularly in patients with relapsed/refractory (R/R) disease. For example, B-cell maturation antigen (BCMA)-directed chimeric antigen receptor T-cell (CAR-T) therapy and T-cell–redirecting bispecific antibodies have improved outcomes across R/R MM (RRMM), including in those with high-risk features. In particular, bringing novel therapies to earlier lines has shown promising results in some patients with HRMM, as seen in CARTITUDE-4 for ciltacabtagene autoleucel (cilta-cel) and MajesTEC-3 for teclistamab plus daratumumab (7–9). The present review focuses on the outcomes of currently approved CAR-T and bispecific T-cell engagers in HRMM and highlights a framework that separates baseline biological and genomic risk, clinical high-risk phenotypes, and context-specific adverse predictors, with particular emphasis on subgroup data in patients with adverse cytogenetics and EMD, the distinct challenge of PCL/circulating plasma cell disease, and emerging targets and next-generation IETs.

For this narrative review, we conducted targeted searches of PubMed/MEDLINE, ClinicalTrials.gov, major hematology and oncology meeting proceedings, and official US Food and Drug Administration resources through August 2026. Search terms included “multiple myeloma,” “high risk,” “functional high risk,” “extramedullary disease,” “plasma cell leukemia,” “CAR-T,” “bispecific antibody,” “BCMA,” “GPRC5D,” and the names of the individual agents discussed. Conference abstracts, retrospective cohorts, and preclinical findings are identified as preliminary where applicable.

## Defining high-risk myeloma

**Table 1** summarizes current high-risk definitions, whereas **Figure 1** contextualizes these criteria within the broader IET-era framework. Historically, high-risk newly diagnosed MM was defined primarily by individual adverse cytogenetic abnormalities such as del(17p), t(4;14), and t(14;16) combined with elevated staging markers. However, as quadruplet induction therapy, autologous stem cell transplantation, and maintenance strategies significantly improved outcomes, single-lesion definitions became inadequate: some patients with high-risk markers achieved durable remissions, while others even with standard-risk relapsed early. This heterogeneity prompted a shift toward integrated, consensus-based risk models, such as the IMS/IMWG consensus criteria, which incorporate del(17p) or *TP53* mutations, chromosome 1 abnormalities, adverse immunoglobulin heavy chain (IgH) translocations, and elevated beta-2 microglobulin (10).

**Table 1:** Proposed IET-era framework for high-risk multiple myeloma.

Domain	Representative features	Interpretation	Practical IET implications
Baseline biological/genomic risk	IMS/IMWG consensus genomic criteria; high-risk transcriptional signatures; plasma-cell S-phase fraction ≥2%	Primarily prognostic; appropriate for baseline stratification and trial balancing	- Does not currently select one IET over another - Combined genomic and aggressive clinical features should prompt early referral and trial consideration
Clinical high-risk phenotypes	Functional HRMM; true non-bone-contiguous EMD; primary or secondary PCL; active circulating plasma cells	Strong adverse prognosis; true EMD must be distinguished from paraskeletal disease, and active PCL from historical PCL	- Assess disease tempo urgently; refer early for IET - Individualize bridging - Prioritize prospective trials
IET-specific adverse predictors	Prior same-target exposure; high tumor burden; elevated ferritin; cytopenias or lymphopenia; poor performance status; impaired immune fitness	Treatment-context prognostic; potentially predictive in selected settings but not validated	- Consider alternate target after same-target exposure - Reduce disease burden when feasible - Avoid delaying referral until further immune deterioration
Feasibility and safety modifiers	Frailty; organ dysfunction; infection risk; hypogammaglobulinemia; access; manufacturing time; caregiver support; patient preference	Modify feasibility and toxicity rather than intrinsic disease biology	- Balance CAR-T vs. bispecific therapy - Supportive care and shared decision-making

EMD, extramedullary disease; HRMM, high-risk multiple myeloma; IET, immune effector therapy; IMS, International Myeloma Society; IMWG, International Myeloma Working Group; MM, multiple myeloma; PCL, plasma cell leukemia.

**Figure 1:**
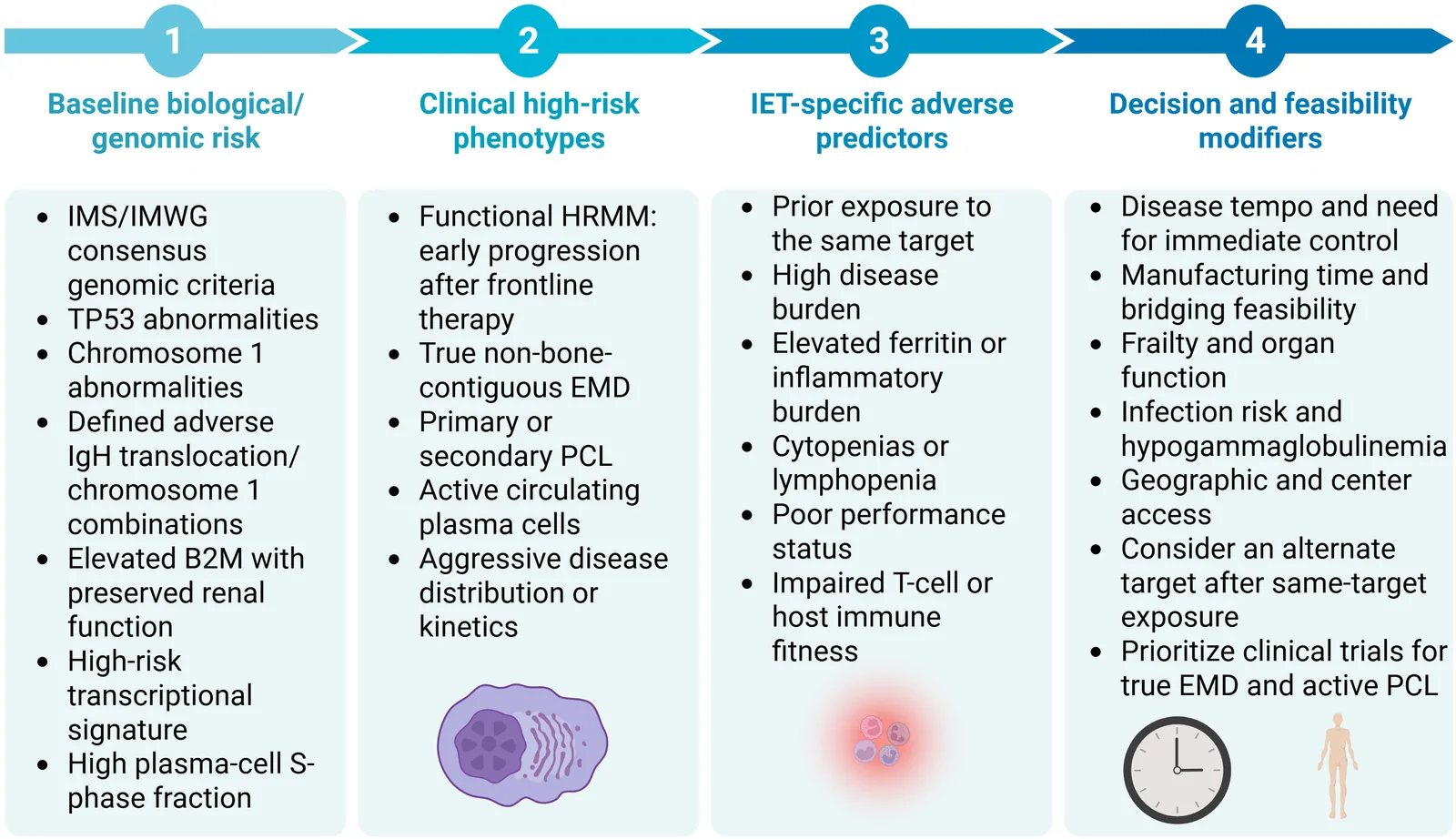
Proposed IET-era framework for high-risk multiple myeloma: risk domains and clinical implications. High-risk disease in the IET era can be assessed across three overlapping domains: baseline biological/genomic risk, clinical high-risk phenotypes, and IET-specific adverse predictors. Frailty, organ function, infection risk, access, manufacturing time, and patient preference modify treatment feasibility. B2M, beta-2 microglobulin; BCMA, B-cell maturation antigen; CAR-T, chimeric antigen receptor T-cell; EMD, extramedullary disease; GPRC5D, G protein–coupled receptor class C group 5 member D; HRMM, high-risk multiple myeloma; IET, immune effector therapy; IgH, immunoglobulin heavy chain; IMS, International Myeloma Society; IMWG, International Myeloma Working Group; PCL, plasma cell leukemia.

For the purposes of this review in the IET era, we organize high-risk features into three distinct domains: (1) baseline biological/genomic risk; (2) clinical high-risk phenotypes; and (3) IET-specific adverse predictors assessed at the time of treatment.

For baseline biological/genomic risk, the IMS/IMWG definition provide a consistent and robust risk stratification (2, 6). Additional baseline biological features include high-risk gene expression profiles and highly proliferative markers (e.g., plasma cell S-phase fraction ≥2%) (11, 12). Clinical high-risk phenotypes include PCL/circulating plasma cell disease and true EMD (13–19). Crucially, true EMD, defined as a soft-tissue plasmacytoma that is not contiguous with bone must be distinguished from paraskeletal disease (direct soft-tissue extension from an osseous lesion), as their underlying biology and prognostic implications differ substantially.

Finally, even in the absence of baseline genomic or phenotypic high-risk markers, early relapse portends a poor prognosis and describes a cohort of patients with “functional” high-risk disease (20–22). The 2025 European Myeloma Network consensus defined functional high-risk MM as disease progression within 18 months of starting first-line therapy in the absence of classical high-risk cytogenetics (1, 5, 6, 23–25). Capturing this aggressive biology, which molecular profiling alone overlooks, is particularly relevant when considering whether IET should be employed earlier in the disease course (26). Separately, although not part of the IMS/IMWG definition, host factors such as frailty, baseline immune function, and barriers to healthcare access should be considered as distinct modifiers of treatment feasibility, intensity, and sequencing.

In the relapsed and refractory (R/R) population treated with IET, clinical phenotypes should be reassessed together with treatment-context–specific adverse predictors. True non-paraskeletal EMD consistently predicts lower response rates and shorter PFS with both CAR-T and bispecific therapies (16).

In real-world cohorts, elevated ferritin, high-disease burden, lymphopenia, and reduced performance status independently portend inferior outcomes, complementing functional high-risk disease defined by relapse within 18 months of first-line therapy (14, 27). The Myeloma CAR-T Relapse (MyCARe) model, developed in a European cohort and externally validated in a United States cohort of patients receiving BCMA CAR-T, assigns one point each to EMD or PCL, lenalidomide refractoriness, high-risk cytogenetics, and elevated ferritin at lymphodepletion. The 5-month incidence of relapse or progression ranged from 7% in the low-risk group to 53% in the high-risk group, illustrating how clinical, disease, and inflammatory variables can inform the timing of cellular therapy (28). Prior BCMA-directed therapy predicts lower response and PFS with subsequent BCMA-targeted agents (23, 29). For patients receiving bispecific T-cell engagers, the externally validated CHARGE Prognostic Index integrates high-risk cytogenetics, ferritin ≥600 ng/mL, hemoglobin <9 g/dL, relapse within 18 months of frontline therapy, ECOG performance status ≥2, and prior BCMA-directed therapy to stratify the risk of early treatment failure (30). For patients proceeding to BCMA-directed CAR-T therapy, the SCOPE-MM score, reported in an IMS abstract, provides a complementary pre-apheresis model for stratifying response and toxicity risk (31). Emerging analyses indicate that both genomic and clinical high-risk features, including EMD, continue to drive inferior outcomes after BCMA-directed bispecific therapy (32). Finally, host immune fitness and the immunosuppressive marrow microenvironment shape IET efficacy, such that comparable biology can yield divergent outcomes; future risk models should incorporate immune phenotype and clonal dynamics alongside tumor genomics.

## Current approvals of CAR-T and bispecific T-cell engagers

As of August 2026, the US FDA-approved MM IETs include two BCMA-directed CAR-T products (idecabtagene vicleucel (ide-cel) and cilta-cel) and four bispecific antibodies, including the BCMA×CD3 agents teclistamab, elranatamab, and linvoseltamab, and the G protein–coupled receptor, family C, group 5, member D (GPRC5D)×CD3 agent talquetamab (**Table 2**).

**Table 2:** Approved immune effector therapies in multiple myeloma.

Agent or regimen	Current US regulatory status/indication	Target/platform	Evidence source and maturity/efficacy signal	High-risk population or definiton	CRS, any grade (grade ≧ 3)	Other notable toxicity/considerations
Idecabtagene vicleucel (ide-cel)	Approved after ≥2 prior lines, including an IMiD, PI, and anti-CD38 monoclonal antibody	BCMA CAR-T	KarMMa-3 (randomized phase 3): mPFS 13.3 vs. 4.4 months vs. standard regimens High-risk cytogenetics: mPFS 11.9 vs. 4.2 mo (HR 0.61); trial-reported extramedullary plasmacytoma HR 0.40	High-risk cytogenetics: del(17p), t(4;14), or t(14;16); marrow plasma cells ≥50%; trial-reported extramedullary plasmacytoma	88% (5%)	Neurotoxic effects 15% (grade ≥3, 3%); grade 3–4 cytopenias; infections
Ciltacabtagene autoleucel (cilta-cel)	Approved after ≥1 prior line including a PI and IMiD, in lenalidomide-refractory disease	BCMA CAR-T	CARTITUDE-1: ORR 97.9%, mPFS 34.9 months CARTITUDE-4 (randomized phase 3): mPFS not reached vs. 11.8 months for standard regimens High-risk cytogenetics: ORR 85% vs. 66%, mPFS 37 vs. 10 mo (HR 0.38); trial-reported EMD mPFS 13 vs. 4 mo	del(17p), t(4;14), t(14;16), or gain/amp(1q); trial-reported EMD	CARTITUDE-1 95% (grade 3–4, 4%; one grade 5 event with HLH) CARTITUDE-4 76.1% (grade 3–4, 1.1%)	CARTITUDE-1 Neurotoxicity 21% (grade 3–4, 9%); prolonged cytopenias; infections CARTITUDE-4 Neurotoxicity 4.5% (all grade 1–2); cranial nerve palsy 9.1%; movement and neurocognitive symptoms 0.6%; CAR-T-related peripheral neuropathy 2.8%
Teclistamab monotherapy	Traditional approval after ≥4 prior lines including a PI, IMiD, and anti-CD38 antibody	BCMA x CD3 bispecific	MajesTEC-1 (phase 1/2): ORR 63.0%, CR or better 39.4%, mPFS 11.3 months ORR 60.5%, CR or better 42.1% for high-risk cytogenetics MajesTEC-9 (randomized phase 3) Overall PFS HR 0.29 vs PVd/Kd	del(17p), t(4;14), or t(14;16)	MajesTEC-1 72.1% (grade 3, 0.6%)	Infections 76.4% (grade 3–4, 44.8%); neutropenia 70.9%; anemia 52.1%
Teclistamab plus daratumumab	Approved after ≥1 prior line including a PI and IMiD		MajesTEC-3 (randomized phase 3): 36-month PFS 83.4% vs. 29.7% with DPd/DVd HR for disease progression or death 0.15 (95% CI 0.09–0.25) for high-risk cytogenetics	ISS stage III and trial-defined high-risk cytogenetics; no dedicated EMD analysis		Infections; hypogammaglobulinemia: cytopenias; CRS; continuous treatment
Elranatamab	Approved after ≥4 prior line including a PI and IMiD	BCMA x CD3 bispecific	MagnetisMM-3 (phase 2): ORR 61.0%, CR rate 35.0%, 15-month PFS rate 50.9% Consistently lower ORR and DOR for high-risk groups; ORR 38.5% and DOR 70.6% (95% CI 56.4–81.0) for EMD, ORR 26.3% and DOR 26.7% (95% CI 1.0–68.6) for R-ISS III, ORR 46.2% and DOR 63.8% (95% CI 37.5–81.3) for penta-refractory disease	t(4;14), t(14;16), or del(17p); R-ISS III; trial-reported EMD; ≥50% marrow plasma cells	57.7% (no grade 3–4)	Infections 69.9% (grade 3–4, 39.8%); neutropenia 48.8%; anemia 48.8%
Linvoseltamab	Accelerated approval after ≥4 prior lines including a PI, IMiD, and anti-CD38 antibody	BCMA x CD3 bispecific	LINKER-MM1 (phase 1/2): ORR 71%, CR rate 50%, 12-month PFS rate 70% at the 200 mg dose High-risk cytogenetics: ORR 67.4%; ISS III 61.9%; non-bone-associated extramedullary plasmacytoma ≥2 cm 52.6%	High-risk cytogenetics; ISS III; non-bone-associated extramedullary plasmacytoma ≥2 cm	46% (grade 3, 1%)	Infections 75% (grade ≥3, 48%); neutropenia 44% (grade ≥3, 43%)
Talquetamab	Accelerated approval after ≥4 prior lines including a PI, IMiD, and anti-CD38 antibody	GPRC5D x CD3 bispecific	MonumenTAL-1 (phase 1/2): ORR 74%, mPFS 6.7 months in high-risk cytogenetics, 8.5 months in standard-risk cytogenetics Trial-reported extramedullary plasmacytoma: mPFS 4.6 vs. 9.2 mo MonumenTAL-3 (randomized phase 3): Tal-DP and Tal-D versus DPd after ≥1 prior line: 24-month PFS, 81.3% and 77.6% vs. 51.2% 24-month OS, 89.2% and 87.9% vs. 79.1%	High-risk cytogenetics and trial-reported extramedullary plasmacytoma	77% at 0.4 mg/kg weekly and 80% at 0.8 mg/kg every other week	Skin-related events 67–70%; dysgeusia 57–63%; nail changes; xerostomia; weight loss

BCMA, B-cell maturation antigen; CD3, cluster of differentiation 3; CR, complete response; CRS, cytokine release syndrome; GPRC5D, G-protein–coupled receptor class C group 5 member D; ORR, overall response rate; PFS, progression-free survival; RRMM, relapsed or refractory multiple myeloma.

Foundational first-in-human BCMA-directed CAR-T studies established proof of concept for deep responses in heavily pretreated MM and preceded the pivotal ide-cel and cilta-cel development programs (33, 34). Teclistamab plus daratumumab was also approved in March 2026 after at least one prior line of therapy, and teclistamab monotherapy was simultaneously converted from accelerated to traditional approval. In HRMM, the question remains how to sequence these novel agents-specifically, whether bispecific antibodies with or without chemoimmunotherapy should precede CAR-T, and which targeted agents should be prioritized.

Throughout this section, subgroup results are drawn from analyses without sufficient powers for the subgroups, with non-uniform definitions of high-risk disease. Given the limitations, where a high-risk subgroup retained a favorable outcome, it should be interpreted as attenuation of the adverse impact of that feature rather than as evidence that it has been overcome by treatment. Also, it is important to recognize that, on current evidence, true EMD with single-agent bispecific antibodies and prior BCMA exposure with subsequent BCMA-directed therapy are predictive, whereas classical high-risk cytogenetics behave predominantly as a prognostic factor.

### Idecabtagene vicleucel

Ide-cel is currently approved for RRMM after two to four prior lines, including a PI, IMiDs, and an anti-CD38 monoclonal antibody. In a pivotal phase 3 KarMMa-3 trial, at the median follow-up of 30.9 months, ide-cel significantly improved progression-free survival (PFS) at 13.3 months (95% CI, 11.8–16.1) vs. 4.4 months (95% CI, 3.4–5.9) for standard regimens, with a hazard ratio (HR) of 0.49 (95% CI, 0.38–0.63; P<0.001). The overall response rate (ORR) was 71% vs. 42% (odds ratio (OR) 3.47; 95% CI, 2.24–5.39; P<0.001), and the complete response (CR) rate was 44% vs 5%, respectively (7, 8). In prespecified subgroup analyses, the PFS benefit with ide-cel was observed across subgroups, including those with high-risk cytogenetic abnormalities [del(17p), t(4;14), t(14;16)], trial-reported extramedullary plasmacytoma, and high tumor burden, although these analyses were not powered for individual subgroups. Median PFS remained superior with ide-cel in high-risk cytogenetics (11.9 vs 4.2 months; HR, 0.61, 95% CI 0.41–0.90), tumor burden ≥50% marrow plasma cells (11.0 vs. 4.9 months; HR 0.60, 95% CI 0.37–0.97), and extramedullary plasmacytoma (HR 0.40, 95% CI 0.25–0.65) (35). The R-ISS III subgroup was small, and the results showed no statistical difference (5.2 vs. 3.0 months; HR 0.86, 95% CI 0.39–1.92). In a subgroup analysis, ide-cel showed ORR/CR rates of 64.5%/31.8% in patients with cytogenetic abnormalities and 55.7%/23.0% in those with trial-reported extramedullary plasmacytoma, vs. 37.7%/4.9% and 18.8%/3.1% with standard regimens, respectively. However, the ORR/CR rates were not statistically superior in the R-ISS III and high-tumor-burden groups.

### Ciltacabtagene autoleucel

Cilta-cel is also approved for RRMM after at least one prior line, including a PI and IMiDs, with lenalidomide-refractory disease. In a phase 1b/2 open-label CARTITUDE-1 study, the ORR was 97.9%, and long-term follow-up showed a median OS of 60.7 months, with approximately one-third of patients remaining progression-free for at least 5 years after a single infusion without maintenance therapy at a median follow-up of 61.3 months (36, 37). The phase 3 CARTITUDE-4 trial confirmed the activity observed in earlier studies and demonstrated superior PFS with cilta-cel versus standard care, supporting its use in earlier relapse (38). Updated analyses showed superior response depth, PFS, and OS compared with standard care in lenalidomide-refractory disease after one to three prior lines, with ORR of 84.6% vs. 67.3%, CR of 73.1% vs. 21.8%, and MRD negativity of 60.6% vs. 15.6%. At a median follow-up of 33.6 months, the results continued to show a survival advantage with median OS not reached in either arm (HR 0.55; 95% CI, 0.39–0.79; P=0.0009) (38). In the CARTITUDE-4 trial, high-risk cytogenetics were defined as del(17p), t(4;14), t(14;16), or gain/amp(1q). In this high-risk subgroup, cilta-cel yielded ORR of 85% vs. 66%, CR or better of 73% vs. 20%, and MRD negativity of 70% vs. 14% compared with standard regimens (39). Patients with high-risk cytogenetics demonstrated a superior median PFS (37 vs. 10 months; HR 0.38, 95% CI 0.27–0.52) and median OS (not reached vs. 38 months; HR 0.54, 95% CI 0.35–0.85) with cilta-cel compared to standard-of-care regimens. It should be noted that these are unplanned subgroup analyses of a small sample size, and the definition of high-risk cytogenetics including gain or amplification of 1q differs from that used in several other trials. By contrast, in patients with trial-reported EMD, the benefits were less pronounced with median PFS of 13 vs. 4 months (HR 0.71, 95% CI 0.34–1.49) and median OS of not reached vs. 16 months (HR 0.61, 95% CI 0.26–1.47) (40).

### Teclistamab

Teclistamab is a BCMA×CD3 bispecific antibody that received accelerated FDA approval in 2022 and full approval in 2026 for RRMM. In a phase 1/2 single-arm MajesTEC-1 trial including 77.6% of triple-class-exposed RRMM, the ORR was 63.0%, the CR rate was 39.4%, and the median PFS was 11.3 months (41). With longer follow-up of 30.4 months, CR or better was 46.1%, median DOR 24.0 months (95% CI, 17.0–not estimable), median PFS 11.4 months (95% CI, 8.8–16.4), and median OS 22.2 months (42). While the subgroup analyses indicate that teclistamab remains active in selected high-risk populations, with patients with high-risk cytogenetics having ORR 60.5% and CR or better 42.1%, shorter median DOR and higher disease-related mortality than in the overall population were noted (43).

The peer-reviewed phase 3 MajesTEC-9 trial randomized 593 patients with RRMM after one to three prior lines, including prior lenalidomide and an anti-CD38 monoclonal antibody, to teclistamab monotherapy or investigator’s choice of PVd or carfilzomib and dexamethasone (44). At a median follow-up of 17.3 months, the estimated 18-month PFS rate was 69.8% with teclistamab versus 26.9% with standard care (HR for progression or death, 0.29; 95% CI, 0.23–0.38). CR or better occurred in 65.9% versus 16.8%, and OS also favored teclistamab (HR for death, 0.60; 95% CI, 0.43–0.83). Grade 3 or 4 infections occurred in 41.6% and 29.0%, respectively. Prespecified subgroup analyses showed a consistent PFS benefit for teclistamab monotherapy over PVd/Kd across all categories, including age, ISS stage, cytogenetic risk, prior lines, and refractoriness. High-risk cytogenetics was defined by the 2016 IMWG triad—del(17p), t(4;14), or t(14;16).

Also, in March 2026, teclistamab plus daratumumab was approved by the FDA for RRMM as early as the second line. In the phase 3 MajesTEC-3 trial comparing teclistamab plus daratumumab with daratumumab, dexamethasone, and either pomalidomide or bortezomib (DPd or DVd), median PFS was not reached with teclistamab-daratumumab vs. 18.1 months (HR 0.17; 95% CI, 0.1–0.23; P<0.0001). Further, the outcomes were consistently superior in the intervention group, with three-year PFS of 83.4% vs. 29.7%, ORR of 89.0% vs. 75.3%, CR or better of 81.8% vs. 32.1%, and MRD negativity of 58.4% vs. 17.1%. OS also favored teclistamab plus daratumumab (HR, 0.46; 95% CI, 0.32–0.65; P<0.0001) (45). In exploratory subgroup analyses, the HR for disease progression or death favored teclistamab plus daratumumab in patients with ISS stage III disease and in those with high-risk cytogenetics defined as the presence of del(17p), t(4;14), or t(14;16), although these subgroups were not powered for formal comparison with wide CIs. Analyses specific to patients with EMD were not reported.

### Elranatamab

Elranatamab provides an additional BCMA off-the-shelf option after at least 4 prior lines of therapy. In a phase 2 single-arm MagnetisMM-3 trial, ORR was 61.0%, with 35.0% achieving CR or better, and 15-month PFS and OS rates being 50.9% and 56.7%, respectively (46). A dedicated high-risk subgroup analysis defined as ‘High cytogenetic risk (t(4;14), t(14;16), or del(17p)),’ ‘R-ISS Stage III,’ ‘Patients with EMD,’ ‘≥50% BMPC,’ were separately reported, and while elranatamab showed efficacy, they were associated with consistently lower ORR and DOR in these high-risk groups compared to those with standard-risk (47). Real-world data from the U.S. Multiple Myeloma Immunotherapy Consortium, which included only 22% of those who would have met the eligibility criteria for MagnetisMM-3, confirmed an ORR of 65% and CR or better in 36% (29).

### Linvoseltamab

Linvoseltamab received accelerated approval in July 2025 after at least 4 prior lines of therapy. In the phase 2 LINKER-MM1 trial, among the larger 200-mg phase 1/2 cohort, ORR was 71% (95% CI, 62–79), CR or better of 52%, and median DOR of 29.4 months. Non-bone-associated extramedullary plasmacytoma ≥2 cm was present in 16% of this cohort. On longer follow-up, responses were observed across high-risk subgroups, with an ORR of 67.4% in high-risk cytogenetics defined as the classic IMWG high-risk triad without incorporating 1q abnormalities, 61.9% in ISS stage III, 66.7% in penta-refractory disease, and 52.6% in the extramedullary subgroup. Response rates in each of these subgroups were nonetheless lower than the 71% observed in the overall 200-mg cohort, and the EMD subgroup remained the least responsive; these are unplanned subgroup analyses of a single-arm study (48).

### Talquetamab

Talquetamab is the first FDA-approved GPRC5DxCD3 bispecific antibody. It is approved for adults with RRMM after at least 4 prior lines, including a PI, an IMiDs, and an anti-CD38 antibody. In the phase 1/2 MonumenTAL-1 trial, ORR was 74% with 0.4 mg/kg weekly dosing, 69% with 0.8mg/kg every-2-week dosing, and 67% in the prior T-cell redirection cohort (49, 50). The trial included 31% with high-risk cytogenetics and 23% with trial-reported extramedullary plasmacytoma in the 0.4 mg/kg weekly dosing population. A post-hoc analysis showed a median PFS of 6.7 months (95% CI, 4.6–11.6) in patients with high-risk cytogenetics defined as the classic IMWG high-risk triad without 1q abnormalities, compared with 8.5 months (95% CI, 6.7–10.9) in standard-risk cytogenetics, and 4.6 months (95% CI, 2.8–5.6) in patients with extramedullary plasmacytoma compared with 9.2 months (95% CI, 7.0–11.8) in those without. In contrast to the BCMA-directed CAR-T data, GPRC5D-directed bispecific monotherapy did not attenuate the adverse impact of either feature.

In the phase 3 MonumenTAL-3 trial for RRMM receiving at least 1 prior line, at time of median follow-up of 24.6 months, talquetamab plus daratumumab and pomalidomide (Tal-DP) and talquetamab plus daratumumab (Tal-D) were associated with longer estimated 24-month PFS over DPd (81.3% and 77.6% vs 51.2%). The intervention groups also had higher ORR (88.2% and 88.5% vs 77.6%), CR or better (71.1% and 69.0% vs 34.5%) and MRD-negativity (52.3% and 46.3% vs 15.9%). 24-month OS estimate with Tal-DP and Tal-D vs DPd was 89.2% (95% CI, 84.9-92.4) and 87.9% (95% CI, 83.0-91.5) vs 79.1% (95% CI, 73.7-83.6) (51). Talquetamab has also shown efficacy as a bridge to BCMA CAR-T without excessive toxicity (52). In this trial, high cytogenetic risk was defined by central or local fluorescence *in situ* hybridization as del(17p), t(4;14), or t(14;16), and was present in 30.9% of patients, although cytogenetic risk was not evaluable in a further 25.7%. The PFS benefit of both talquetamab-containing regimens over DPd appeared consistent in the prespecified high-cytogenetic-risk subgroup. Also, true extramedullary plasmacytoma, defined as a plasmacytoma not contiguous with bone, was distinguished from paraskeletal disease at baseline, with 15.0% of patients having at least one soft-tissue plasmacytoma, among whom 36.9% had true extramedullary and 77.7% paraskeletal involvement. While talquetamab shows efficacy in patients with high-risk cytogenetics, it carries distinct on-target toxicities, including dysgeusia, xerostomia, skin and nail changes, and weight loss, which affect tolerability (49).

## Outcomes of IET in plasma cell leukemia

The IMWG defines PCL by the presence of ≥5% circulating plasma cells on a conventional peripheral blood smear, a threshold lowered in 2021 from the historical ≥20% (or >2×10^9^/L) once outcomes proved similarly poor above 5%. Primary PCL arises *de novo*, whereas secondary PCL represents leukemic transformation of MM. PCL is a subset of HRMM associated with worse outcomes; in a recent institutional series, both primary and secondary PCL showed markedly inferior survival relative to other extramedullary presentations (53). The evidence base should distinguish primary versus secondary PCL because these populations are biologically and clinically different. Patients with PCL were generally excluded from pivotal IET trials, and most available evidence is retrospective, heterogeneous, and based on small cohorts. Direct comparisons among products should therefore be interpreted with cautions.

Patients with PCL often carry adverse genetics such as MAF/MAFB translocations, 1q gain, and del(17p), high tumor burden, and a high rate of extramedullary involvement. These patients were largely excluded from pivotal IET trials, and the level of evidence in this area remains sparse. The evidence related to PCL largely comes from real-world data (54).

A multicenter retrospective study of CAR-T therapy evaluated 34 patients with either pPCL or sPCL who received BCMA CAR-T (19 with ide-cel and 15 with cilta-cel). At a median follow-up of 11.9 months, the median PFS was 9.0 months (95% CI, 4–15) and the median OS was 13.0 months (95% CI, 8–not estimable). Outcomes were numerically better with cilta-cel than ide-cel, with median PFS 19.0 versus 6.0 months, median OS >23 versus 9.0 months, and 1-year progression/death incidence 37.5% (95% CI 17.4–68.5) with cilta-cel, whereas all ide-cel-treated patients progressed or died within 12 months (55). A separate retrospective cohort included 26 patients with pPCL or sPCL treated with ide-cel (n = 8) or cilta-cel (n = 18). Numerically higher ORR and early MRD-negativity rates were observed with cilta-cel; however, the small, nonrandomized groups preclude conclusions regarding comparative superiority. Best ORR was 94% versus 63%, and 1-month MRD negativity was 80% versus 38%, respectively. Median PFS was not reached in the cilta-cel group and was 5.4 months in the ide-cel group (56).

For bispecific antibodies, a multicenter retrospective study evaluated the outcomes of 16 patients who received teclistamab in secondary PCL at three U.S. academic centers. All 16 patients had high-risk cytogenetics and 31% also had soft-tissue EMD. ORR was 31%, with one CR and four PRs. The median PFS for the entire cohort was 27 days (95% CI, 12–85), and none of the prior BCMA-exposed patients responded, with a median PFS of 4 days. The largest bispecific antibody dataset in PCL was reported from the U.S. Myeloma consortium, including 122 patients (57). The study included four cohorts; 37% with teclistamab, 11% with elranatamab, 42% with talquetamab definitive treatment, and 11% with talquetamab bridging to CAR-T. Overall, ORR was 54.5%, with median PFS of 3.2 months and median OS of 9.2 months. Among the agents, talquetamab demonstrated the highest numerical outcomes, with ORR 60.8%, median PFS 5.5 months, and median OS 11.5 months; teclistamab had median PFS 1.2 months and median OS 8.1 months; and elranatamab had median PFS 1.6 months and median OS 3.6 months. In active PCL, multivariable analysis showed that talquetamab was associated with better PFS (HR 0.41, 95% CI 0.21–0.81; P=0.009) and OS (HR 0.40, 95% CI 0.19–0.82; P=0.012).

PCL remains a major therapeutic challenge and is associated with worse outcomes than most other HRMM phenotypes. BCMA-directed CAR-T therapy and talquetamab may provide activity in patients with active PCL or circulating disease, although the evidence is retrospective and future studies are warranted to verify the findings with a prospective cohort. Preliminary prospective evidence in newly diagnosed primary PCL has been reported as conference abstracts. The single-arm CAREMM-002 study treated transplant-ineligible patients with VRd-based induction followed by BCMA CAR-T, reporting an ORR of 100% with frequent MRD-negative stringent complete responses and no early relapses (58). A sequential CAR-T–ASCT–CAR-T (“sandwich”) regimen likewise produced deep, MRD-negative responses in newly diagnosed primary PCL (59). These early prospective signals provide a rationale for studying frontline IET in primary PCL, but they remain promising and unconfirmed pending larger prospective trials with durable follow-up. A 2026 systematic review of CAR-T therapy across primary and secondary PCL similarly concluded that BCMA-directed CAR-T can produce deep responses, while underscoring limited durability and a lack of prospective data (60). Outcomes in PCL must be interpreted alongside other adverse risk features. True EMD remains strongly prognostic: in a real-world teclistamab analysis of 385 patients, true-EMD involvement carried an ORR of 38% and median PFS of 1.4 months, versus 62.4% and 8.95 months without soft-tissue plasmacytomas (61).

## Outcomes with IET in renal impairment

Renal impairment can affect a substantial proportion of patients with MM. However, it is commonly an exclusion criterion in most registrational IET studies. In a multicenter analysis of 384 patients treated with standard-of-care teclistamab, 21% had renal impairment (defined as creatinine clearance <40 mL/min), including 5% on dialysis. The response rates and safety were comparable to patients with preserved renal function, while those with renal dysfunction had more cytopenias (62). Prior BCMA-directed therapy has become one of the most notable adverse features in relapsed disease. In an IMWG real-world teclistamab study, prior BCMA exposure was associated with a lower ORR (58% vs. 74%) and shorter 6-month PFS (43% vs. 63%) (63). By contrast, GPRC5D-directed agents retain meaningful activity after BCMA exposure, with talquetamab achieving a 67% ORR in the prior–T-cell-redirection cohort of MonumenTAL-1 and serving effectively as a bridge to BCMA CAR-T (49, 52).

## Clinical implementation and patient-centered outcomes

CAR-T therapy offers time-limited treatment but requires referral, leukapheresis, manufacturing, lymphodepletion, and frequently bridging therapy, and patients with rapidly progressive HRMM may clinically deteriorate before infusion (64). Bispecific antibodies are immediately available but generally require ongoing dosing and are also associated with CRS, infections, hypogammaglobulinemia, and cytopenias (65). Prolonged cytopenias and infection risk also remain important after CAR-T therapy. Frailty, organ dysfunction, performance status, caregiver support, geographic access, center capacity, and patient preference therefore modify treatment feasibility and safety. Patient-reported outcomes support these clinical gains. Health-related quality of life improved with cilta-cel compared with standard of care in CARTITUDE-4 and with ide-cel compared with standard regimens in KarMMa-3, and symptom and functional scores improved or were maintained with other IETs in their respective studies (66–68). However, patients with true EMD, active PCL, severe frailty, and major organ dysfunction remain underrepresented in these trials.

Implementation is also constrained by cost. Model-based analyses indicate that the value of anti-BCMA CAR-T depends heavily on durability of response. Assuming a 5-year PFS of 40% with cilta-cel, the incremental cost-effectiveness ratio versus a no-CAR-T strategy was $112,191 per quality-adjusted life-year (QALY) in the United States, below a $150,000 willingness-to-pay threshold (69). Earlier-line use of the most effective agents may therefore be more cost-effective than late-line use, although head-to-head economic comparisons with bispecific antibodies are lacking (70).

## Biological mechanisms and biomarkers of IET failure

Resistance to IET arises from an interplay among target-antigen escape, T-cell dysfunction, and an immunosuppressive tumor microenvironment that frequently coexist. Target-driven resistance can arise through genomic alterations, including monoallelic deletions followed by acquisition or selection of a second hit at *TNFRSF17* (16p) or *GPRC5D* (12p), as well as through epitope-altering mutations, transcriptional or epigenetic downregulation, and other mechanisms of antigen modulation (5, 71, 72). Heterozygous deletions of TNFRSF17 and GPRC5D are detectable before any T-cell–redirecting therapy and may represent a first hit predisposing to antigen-loss relapse (73). After GPRC5D-directed bispecific therapy, resistance has been attributed to biallelic genetic inactivation or long-range epigenetic silencing of GPRC5D (74). BCMA is additionally regulated by γ-secretase-mediated cleavage, which decreases surface BCMA density and generates soluble BCMA (sBCMA); high sBCMA can act as a decoy and impair the activity of BCMA-directed bispecific antibodies (75). On the effector side, t nadequate T-cell fitness, impaired expansion and persistence, and progressive exhaustion characterized by expression of inhibitory receptors including PD-1, TIM-3, and LAG-3 can limit efficacy (76). For example, an abundance of exhausted CD8+ T-cell clones and loss of MHC class I are associated with failure of bispecific T-cell engagers, whereas reduced pre-therapy T-cell receptor diversity, hyperexpanded exhausted clones, and BAFF+PD-L1+ myeloid cells in the bone marrow predict shorter PFS after BCMA CAR-T (77). Because cumulative exposure to chemoimmunotherapy and prior T-cell redirection erodes this compartment, effector fitness is itself a determinant of outcome and an argument for earlier deployment of IET.

## Emerging targets and next-generation immune effector therapies

Resistance to BCMA- or GPRC5D-directed therapy can arise through biallelic target loss or mutation, heterogeneous target expression, branching clonal evolution, inadequate CAR-T persistence, and progressive T-cell dysfunction (5). Dual-targeting strategies are intended to reduce single-antigen escape, whereas alternative targets may preserve activity after prior BCMA exposure. Here, we will introduce representative emerging targets and next-generation IETs for HRMM (**Table 3**). The evidence summarized spans peer-reviewed early-phase clinical studies and preclinical work, with each identified accordingly.

**Table 3:** Emerging immune effector strategies and maturity of supporting evidence.

Target and/or platform	Representative agents	Use for HRMM	Evidence maturiaty	Principal limitation
GPRC5D CAR-T	MCARH109 RD118 Arlocabtagene autoleucel (arlo-cel)	Option following BCMA-directed therapy failure	Peer-reviewed phase 1	Small nonrandomized cohorts; limited durability and comparative data
Trispecific antibodies	Ramantamig (BCMA/GPRC5D/CD3) IBI3003 (BCMA/GPRC5D/CD3) ISB2001 (BCMA/CD38/CD3)	Designed to reduce single-antigen escape and better address clonal heterogeneity	Phase 1 conference abstracts; preclinical evidence varies by agent	Preliminary efficacy; limited follow-up; no randomized HRMM evidence
CD70 CAR-T	N/A	Highly expressed in HRMM	Preclinical only	No clinical efficacy or safety data
FcRH5 x CD3 bispecific antibodies	Cevostamab	Non-BCMA or GPRC5D salvage option; may be useful after BCMA- or GPRC5D-directed therapy failure	Peer-reviewed phase 1/2	No validated HRMM-specific benefit

BCMA, B-cell maturation antigen; CD3, cluster of differentiation 3; CD38, cluster of differentiation 38; CD70, cluster of differentiation 70; GPRC5D, G-protein–coupled receptor class C group 5 member D; FcRH5, Fc receptor-homolog 5.

### Dual antigen targeting

One response to treatment failure after BCMA or GPRC5D targeted therapy is dual-antigen targeting. In the phase 2 RedirecTT-1 trial, a prespecified cohort of patients with prespecified true EMD received talquetamab plus teclistamab. The ORR was 78.9%, and among responders, 64% (95% CI, 48–76) maintained a response for at least 12 months. The 12-month PFS and OS rates were 61% (95% CI, 50–71) and 74% (95% CI 63–83), respectively. Because true EMD has been associated with poor outcomes after single-agent BCMA- or GPRC5D-directed therapy, these results support further evaluation of dual-target treatment in this population (78). Talquetamab plus daratumumab (phase 1b TRIMM-2) produced an ORR of 71.4% in the weekly cohort and 82.4% in the every-other-week cohort, with median PFS of 23.3 and 21.2 months, respectively, and retained activity after prior bispecific exposure (79).

### GPRC5D CAR-T

GPRC5D-directed CAR-T therapies have shown high efficacy. The phase 1 first-in-human study of MCARH109, GPRC5D-targeted CAR-T, demonstrated a 71% response rate in 17 heavily pretreated patients, including 70% with prior BCMA-targeted therapy (80, 81). Further, in a phase 1 trial of RD118, fully human single-domain anti-GPRC5D CAR-T including 17 RRMM and primary PCL, the ORR was 94.4%, with 72.2% CR/sCR, median PFS of 18.2 months (95% CI, 14.4–not estimable), and 12-month PFS and OS rates of 82.1% and 93.3%, respectively. Among seven patients with prior BCMA CAR-T exposure, the ORR was 85.7% (82). Finally, a phase 1 dose escalation/expansion study of the GPRC5D CAR-T arlocabtagene autoleucel (arlo-cel) included 84 RRMM patients with at least three prior lines. The trial demonstrated ORR of 87%, CR rate of 53%, and median PFS of 18.3 months (95% CI, 11.8–21.9) (83). In patients who had received prior BCMA-targeted therapy, ORR was 79%, compared with 95% among BCMA-naïve patients. ORRs were 84% and 90% among patients with and without high-risk cytogenetics, respectively. These early-phase results support GPRC5D-directed CAR-T as a potential strategy after BCMA-directed therapy, although longer follow-up and prospective comparative data are needed.

### Trispecific antibodies

Trispecific antibodies have been explored as a potential agent to improve outcomes in HRMM. Ramantamig (JNJ-79635322), a BCMA/GPRC5D/CD3 trispecific antibody, has demonstrated dual-antigen activity in preclinical models. Preliminary phase 1 results were subsequently reported in a 2025 conference abstract: at the putative recommended phase 2 dose, the ORR was 86%, including 100% among the 27 patients who were naïve to both BCMA- and GPRC5D-directed therapy (84). These early clinical findings require longer follow-up and peer-reviewed confirmation. The study is ongoing (NCT05652335), and the efficacy of combination therapy with ramantamig with daratumumab or IMiDs has been explored (NCT06768489). IBI3003 is another novel trispecific antibody targeting BCMA/GPRC5D/CD3 and has shown encouraging preliminary data in RRMM. Among patients treated at ≥120 μg/kg, ORR was 84.6%. Of note, in patients with EMD, ORR was notable at 83.3% (85). ISB2001 is a BCMA/CD38/CD3 trispecific antibody that has been explored. In the phase 1 dataset, ORR was 74% overall and 79% across active dose levels in RRMM, including 71% being penta-exposed (86).

### Fc receptor-homolog 5

Among targets beyond BCMA or GPRC5D, Fc receptor-homolog 5 (FcRH5) is one of the notable emerging targets. FcRH5 is a type I membrane protein exclusively expressed in the B-cell lineage and found at a higher level on MM cells than on normal B cells. Cevostamab is an FcRH5×CD3 bispecific antibody with the most mature data, demonstrating an ORR of 43.1% and a median DOR of 10.4 months (95% CI, 6.8–10.5) among 167 RRMM patients, including 57% who had received ≥1 prior BCMA-targeted therapy with acceptable side effect profiles (87).

### CD70

CD70 (CD27L/TNFSF7) is a type II transmembrane glycoprotein and TNF superfamily member (88). While its expression is restricted to activated T and B lymphocytes and mature dendritic cells, CD70 is aberrantly expressed on approximately 40% of MM (89). Preclinical studies suggest that CD70 may be enriched in selected high-risk MM genotypes and may represent a therapeutic target (90). However, no clinical efficacy or safety data for CD70-directed therapy in MM are currently available, and restricted expression in normal tissues does not establish the absence of off-tumor toxicity.

In preclinical models, optimized anti-CD70 CAR-T cells expanded *in vivo*, and dual CD70/BCMA targeting reduced antigen-escape–mediated resistance. These results remain preclinical and should be considered hypothesis-generating (90).

## Conclusion

Treatment strategies for RRMM have advanced substantially with the introduction of BCMA- and GPRC5D-targeted therapies, but durable control of high-risk disease remains the central unmet need, particularly in true EMD, PCL, and disease relapsing after BCMA- or GPRC5D-directed therapy. The evidence reviewed here indicates that the IET era has changed how we define high-risk categories; classical high-risk cytogenetics are attenuated, whereas anatomic and immunologic features have become the dominant determinants of benefit. High-risk status should be assessed across all three domains including genomic, clinical phenotype, and IET-specific factors, rather than by cytogenetics alone. Patients with true EMD, PCL, prior BCMA exposure, or organ dysfunction, who are systematically under-represented in registrational trials, should be prioritized for trials of next-generation IETs. Research priorities include prospective risk-stratified trials, validation of genomic and immune biomarkers, direct sequencing studies, and routine incorporation of patient-reported outcomes, infection burden, access, and cost. These efforts are required to enable convert predictive treatment selection.

## References

[1] Lim SL Engelhardt M Terpos E Gay F Van de Donk N Einsele H . European myeloma network consensus statement on functional high-risk multiple myeloma. Am J Hematol. (2025) 100:2320–32. doi: 10.1002/ajh.70070 40926508

[2] Kastritis E Gavriatopoulou M Theodorakakou F Solia E Ntanasis-Stathopoulos I Malandrakis P . Validation of the new ims/IMWG consensus genomic staging (CGS) of high-risk multiple myeloma (HRMM) in a contemporary cohort of 1209 patients. Blood. (2025) 146:923. doi: 10.1182/blood-2025-923 PMC13446282 42565138

[3] Rodriguez-Otero P Usmani S Cohen AD van de Donk N Leleu X Gállego Pérez-Larraya J . International myeloma working group immunotherapy committee consensus guidelines and recommendations for optimal use of T-cell-engaging bispecific antibodies in multiple myeloma. Lancet Oncol. (2024) 25:e205–16. doi: 10.1016/s1470-2045(24)00043-3 38697166

[4] Lin Y Qiu L Usmani S Joo CW Costa L Derman B . Consensus guidelines and recommendations for the management and response assessment of chimeric antigen receptor T-cell therapy in clinical practice for relapsed and refractory multiple myeloma: a report from the International Myeloma Working Group Immunotherapy Committee. Lancet Oncol. (2024) 25:e374–87. doi: 10.1016/s1470-2045(24)00094-9 38821074

[5] Lee H Ahn S Maity R Leblay N Ziccheddu B Truger M . Mechanisms of antigen escape from BCMA- or GPRC5D-targeted immunotherapies in multiple myeloma. Nat Med. (2023) 29:2295–306. doi: 10.1038/s41591-023-02491-5 37653344 PMC10504087

[6] Hanamura I . Multiple myeloma with high-risk cytogenetics and its treatment approach. Int J Hematol. (2022) 115:762–77. doi: 10.1007/s12185-022-03353-5 35534749 PMC9160142

[7] Rodriguez-Otero P Ailawadhi S Arnulf B Patel K Cavo M Nooka AK . Ide-cel or standard regimens in relapsed and refractory multiple myeloma. N Engl J Med. (2023) 388:1002–14. doi: 10.1056/nejmoa2213614 36762851

[8] Ailawadhi S Arnulf B Patel K Cavo M Nooka AK Manier S . Ide-cel vs standard regimens in triple-class-exposed relapsed and refractory multiple myeloma: updated KarMMa-3 analyses. Blood. (2024) 144:2389–401. doi: 10.1182/blood.2024024582 39197072

[9] Munshi NC Anderson LD Shah N Madduri D Berdeja J Lonial S . Idecabtagene vicleucel in relapsed and refractory multiple myeloma. N Engl J Med. (2021) 384:705–16. doi: 10.1056/nejmoa2024850 33626253

[10] Avet-Loiseau H Davies FE Samur MK Corre J D'Agostino M Kaiser MF . International myeloma society/International myeloma working group consensus recommendations on the definition of high-risk multiple myeloma. J Clin Oncol. (2025) 43:2739–51. doi: 10.1200/jco-24-01893 40489728 PMC13445576

[11] McAvera R Cymer I McAuley N Badar S O'Neill G Murphy PT . Identification of drivers of SKY92 high-risk multiple myeloma. Blood. (2024) 144:5771. doi: 10.1182/blood-2024-209762

[12] Beer SA Cairns DA Pawlyn C Holroyd A Ferris E Cook G . Challenging the concept of functional high-risk myeloma through transcriptional and genetic profiling. Blood. (2025) 146:2670–80. doi: 10.1182/blood.2025029987 40834881 PMC12824701

[13] Zanwar S Ho M Lin Y Kapoor P Binder M Buadi FK . Natural history, predictors of development of extramedullary disease, and treatment outcomes for patients with extramedullary multiple myeloma. Am J Hematol. (2023) 98:1540–9. doi: 10.1002/ajh.27023 37421603

[14] Riedhammer C Bassermann F Besemer B Bewarder M Brunner F Carpinteiro A . Real-world analysis of teclistamab in 123 RRMM patients from Germany. Leukemia. (2024) 38:365–71. doi: 10.1038/s41375-024-02154-5 38245601 PMC10844072

[15] Dima D Abdallah AO Davis JA Awada H Goel U Rashid A . Impact of extraosseous extramedullary disease on outcomes of patients with relapsed-refractory multiple myeloma receiving standard-of-care chimeric antigen receptor T-cell therapy. Blood Cancer J. (2024) 14:90. doi: 10.1038/s41408-024-01068-w 38821914 PMC11143360

[16] Zanwar S Sidana S Shune L Puglianini OC Pasvolsky O Gonzalez R . Impact of extramedullary multiple myeloma on outcomes with idecabtagene vicleucel. J Hematol Oncol. (2024) 17:42. doi: 10.1186/s13045-024-01555-4 38845015 PMC11157748

[17] Zanwar S Jevremovic D Kapoor P Olteanu H Buadi F Horna P . Clonal plasma cell proportion in the synthetic phase identifies a unique high-risk cohort in multiple myeloma. Blood Cancer J. (2025) 15:20. doi: 10.1038/s41408-025-01232-w 39966346 PMC11836398

[18] Hellou T Packard DG Atallah-Yunes SA Orusa AM Suzuki S Kumar SK . Impact of residual clonal plasma cells in S-phase at the time of autologous stem cell transplantation on clinical outcomes. Blood Cancer J. (2025) 15:152. doi: 10.1038/s41408-025-01349-y 41022708 PMC12480884

[19] Bertamini L Oliva S Rota-Scalabrini D Paris L Morè S Corradini P . High levels of circulating tumor plasma cells as a key hallmark of aggressive disease in transplant-eligible patients with newly diagnosed multiple myeloma. J Clin Oncol. (2022) 40:3120–31. doi: 10.1200/jco.21.01393 35666982

[20] Kumar SK Dispenzieri A Fraser R Mingwei F Akpek G Cornell R . Early relapse after autologous hematopoietic cell transplantation remains a poor prognostic factor in multiple myeloma but outcomes have improved over time. Leukemia. (2018) 32:986–95. doi: 10.1038/leu.2017.331 29263438 PMC5871538

[21] Goel U Dragomirescu C Zanwar S Cassano RC Cicero KI Cowan AJ . Functional high-risk phenotype predicts poor survival in multiple myeloma independent of front-line treatment: a secondary analysis of CIBMTR data. Br J Haematol. (2026) 208:2134–42. doi: 10.53347/rid-28007 PMC13267472 42023513

[22] Jimenez-Zepeda VH Reece DE Trudel S Chen C Tiedemann R Kukreti V . Early relapse after single auto-SCT for multiple myeloma is a major predictor of survival in the era of novel agents. Bone Marrow Transplant. (2015) 50:204–8. doi: 10.1038/bmt.2014.237 25347006

[23] Sidana S Ahmed N Akhtar OS Brazauskas R Oloyede T Bye M . Standard-of-care idecabtagene vicleucel for relapsed/refractory multiple myeloma. Blood. (2025) 146:167–77. doi: 10.1182/blood.2024026216 40198886 PMC12964216

[24] Costa LJ Weisel KC Donk N Sidana S Cohen YC Purtill D . Ciltacabtagene autoleucel vs standard of care in patients with functional high-risk multiple myeloma: CARTITUDE-4 subgroup analysis. J Clin Oncol. (2024) 42:7504. doi: 10.1016/s2152-2650(24)00989-3

[25] Morè S Offidani M Corvatta L Za T Fazio F Gherardini M . P65 | Functional high risk multiple myeloma in the anti-CD38 quadruplet era: Redefinition through real world data. Haematologica. (2026) 111.

[26] Banerjee R Cicero KI Lee SS Cowan AJ . Definers and drivers of functional high-risk multiple myeloma: insights from genomic, transcriptomic, and immune profiling. Front Oncol. (2023) 13:1240966. doi: 10.3389/fonc.2023.1240966 37849816 PMC10577204

[27] Razzo BM Midha S Portuguese AJ Grajales-Cruz AF De Menezes Silva Corraes A Costello P . Real-world experience with teclistamab for relapsed/refractory multiple myeloma from the US Myeloma Immunotherapy Consortium. Blood Cancer Discov. (2025) 6:561–71. doi: 10.1158/2643-3230.bcd-24-0354 40629516

[28] Gagelmann N Dima D Merz M Hashmi H Ahmed N Tovar N . Development and validation of a prediction model of outcome after B-cell maturation antigen-directed chimeric antigen receptor T-cell therapy in relapsed/refractory multiple myeloma. J Clin Oncol. (2024) 42:1665–75. doi: 10.1200/jco.23.02232 38358946 PMC11095856

[29] Portuguese AJ Davis JA Raza S Castaneda Puglianini O Freeman C Alsina M . Real-world outcomes with elranatamab in multiple myeloma: a multicenter analysis from the U.S. Multiple Myeloma Immunotherapy Consortium. Blood Cancer J. (2026) 16:47. doi: 10.1038/s41408-026-01477-z 41896552 PMC13039266

[30] Zanwar S Hansen D Pasvolsky O Tan C Peres L Goel U . Early treatment failure in patients with relapsed/refractory multiple myeloma receiving bispecific T-cell engagers: development and validation of the CHARGE prognostic index. J Clin Oncol. (2026), In press.

[31] Cordas dos Santos DM Rejeski K Geyer SM Dooley K Tix T Bassermann F . (2025). Development and validation of the SCOPE-MM score: a pre-apheresis risk model predicting response and toxicity in RRMM patients receiving BCMA-directed CAR-T therapy. Clin Lymphoma Myeloma Leuk. (2025) 25(Suppl 2):S2–3. Abstract OA-03.

[32] Longobardi S Oo SL Shrestha A Trikannad AK Vellanki S Cheema HI . Influence of genomic and clinical high-risk features on outcomes after BCMA-directed bispecific antibody therapy in relapsed/refractory multiple myeloma. J Clin Oncol. (2026) 44:7534. doi: 10.1200/jco.2026.44.16_suppl.753442697810

[33] Ali SA Shi V Maric I Wang M Stroncek DF Rose JJ . T cells expressing an anti–B-cell maturation antigen chimeric antigen receptor cause remissions of multiple myeloma. Blood. (2016) 128:1688–700. doi: 10.1182/blood-2016-04-711903 27412889 PMC5043125

[34] Raje N Berdeja J Lin Y Siegel D Jagannath S Madduri D . Anti-BCMA CAR T-cell therapy bb2121 in relapsed or refractory multiple myeloma. N Engl J Med. (2019) 380:1726–37. doi: 10.1056/nejmoa1817226 31042825 PMC8202968

[35] Patel K Rodríguez-Otero P Manier S Baz R Raab MS Cavo M . S195: Idecabtagene vicleucel (ide-cel) vs standard regimens in patients with triple-class–exposed (TCE) relapsed and refractory multiple myeloma (RRMM): a KarMMa-3 analysis in high-risk subgroups. Hemasphere. (2023) 7(S3):e369897b. doi: 10.1097/01.hs9.0000967692.36989.7b33079766

[36] Martin T Usmani SZ Berdeja JG Agha M Cohen AD Hari P . Ciltacabtagene autoleucel, an anti-B-cell maturation antigen chimeric antigen receptor T-cell therapy, for relapsed/refractory multiple myeloma: CARTITUDE-1 2-year follow-up. J Clin Oncol. (2023) 41:1265–74. doi: 10.1200/jco.22.00842 35658469 PMC9937098

[37] Jagannath S Martin TG Lin Y Cohen AD Raje N Htut M . Long-term (≥5-year) remission and survival after treatment with ciltacabtagene autoleucel in CARTITUDE-1 patients with relapsed/refractory multiple myeloma. J Clin Oncol. (2025) 43:2766–71. doi: 10.1200/jco-25-00760 40459151 PMC12393059

[38] Einsele H San-Miguel J Dhakal B Touzeau C Leleu X van de Donk NW . Cilta-cel in lenalidomide-refractory multiple myeloma (CARTITUDE-4): an updated analysis including overall survival from an open-label, multicentre, randomised, phase 3 trial. Lancet Oncol. (2026) 27:254–68. doi: 10.1016/s1470-2045(25)00653-9 41519141

[39] Mina R Dhakal B San-Miguel J Lendvai N Vogel M Li K . Ciltacabtagene autoleucel vs standard of care in lenalidomide-refractory multiple myeloma: phase 3 CARTITUDE-4 subgroup analysis by cytogenetic risk. Hematology Transfusion Cell Ther. (2024) 46:S533–4. doi: 10.1016/j.htct.2024.09.89542574925

[40] Sidana S Martínez-López J Khan A Oriol A Spencer A Dhakal B . MM-1070: Ciltacabtagene autoleucel vs standard of care in patients with relapsed/refractory multiple myeloma: CARTITUDE-4 survival subgroup analyses. Clin Lymphoma Myeloma Leukemia. (2025) 25:S963–4. doi: 10.1016/s2152-2650(25)02682-531792650

[41] Moreau P Garfall AL van de Donk N Nahi H San-Miguel JF Oriol A . Teclistamab in relapsed or refractory multiple myeloma. N Engl J Med. (2022) 387:495–505. doi: 10.1056/nejmoa2203478 35661166 PMC10587778

[42] Garfall AL Nooka AK Donk N Moreau P Bhutani M Oriol A . Long-term follow-up from the phase 1/2 MajesTEC-1 trial of teclistamab in patients with relapsed/refractory multiple myeloma. J Clin Oncol. (2024) 42:7540. doi: 10.1016/s2152-2650(24)00953-4

[43] Costa LJ Bahlis NJ Usmani SZ van de Donk NWCJ Nooka AK Perrot A . MM-328 efficacy and safety of teclistamab in patients with relapsed/refractory multiple myeloma (RRMM) with high-risk (HR) features: a subgroup analysis from the phase 1/2 MajesTEC-1 study. Clin Lymphoma Myeloma Leukemia. (2024) 24:S546–7. doi: 10.1016/s2152-2650(24)01664-1

[44] Touzeau C Mina R Quach H Hungria V Bhutani D Chen W . Teclistamab in multiple myeloma with one to three previous lines of therapy. N Engl J Med. (2026) 395:440–53. doi: 10.1056/nejmoa2603870 42212933

[45] Costa LJ Bahlis NJ Perrot A Nooka AK Lu J Pawlyn C . Teclistamab plus daratumumab in relapsed or refractory multiple myeloma. N Engl J Med. (2026) 394:739–52. doi: 10.1056/nejmoa2514663 41363801 PMC13218738

[46] Lesokhin AM Tomasson MH Arnulf B Bahlis NJ Miles Prince H Niesvizky R . Elranatamab in relapsed or refractory multiple myeloma: phase 2 MagnetisMM-3 trial results. Nat Med. (2023) 29:2259–67. doi: 10.1038/s41591-023-02528-9 37582952 PMC10504075

[47] Touzeau C Bahlis NJ Maisel C Karlin L Nina Varshavsky-Yanovsky A Leip E . P907: Efficacy and safety of elranatamab in high-risk patients with relapsed/refractory multiple myeloma: a subgroup analysis from MagnetisMM-3. Hemasphere. (2023) 7(S3):e16622f0. doi: 10.1097/01.hs9.0000970532.16622.f033079766

[48] Lee HC Zonder JA Dhodapkar MV Jagannath S Hoffman JE Suvannasankha A . Linvoseltamab in patients with relapsed/refractory multiple myeloma in the LINKER-MM1 study: longer follow-up and subgroup analyses. Clin Lymphoma Myeloma Leukemia. (2026) 26:e201–e212.e8. doi: 10.1016/j.clml.2025.11.004 41387038

[49] Chari A Touzeau C Schinke C Minnema MC Berdeja JG Oriol A . Safety and activity of talquetamab in patients with relapsed or refractory multiple myeloma (MonumenTAL-1): a multicentre, open-label, phase 1–2 study. Lancet Haematology. (2025) 12:e269–81. doi: 10.1016/s2352-3026(24)00385-5 40090350

[50] Chari A Minnema MC Berdeja JG Oriol A Donk N Rodríguez-Otero P . Talquetamab, a T-cell–redirecting GPRC5D bispecific antibody for multiple myeloma. N Engl J Med. (2022) 387:2232–44. doi: 10.1056/nejmoa2204591 36507686

[51] Mina R Beksac M Rodríguez-Otero P Chen W Mateos M-V Li J . Talquetamab–daratumumab in relapsed or refractory myeloma. N Engl J Med. (2026) 395:671–83. doi: 10.1056/nejmoa2604657 42294841

[52] Dhakal B Akhtar OS Fandrei D Jensen A Banerjee R Pan D . Sequential targeting in multiple myeloma: talquetamab, a GPRC5D bispecific antibody, as a bridge to BCMA CAR-T therapy. Blood. (2025) 146:2063–72. doi: 10.1182/blood.2025029773 40749169

[53] Broughton M Bhatta S Sonali D Bumma N Khan AM Devarakonda S . Comprehensive evaluation of disease characteristics and outcomes of patients with extramedullary multiple myeloma in the modern era. Haematologica. (2025) 110:3053–64. doi: 10.3324/haematol.2025.287545 40501390 PMC12666302

[54] Hanamura I Karnan S Ota A Takami A . Primary plasma cell leukemia: recent advances in molecular understanding and treatment approaches. Int J Mol Sci. (2025) 26(13):6166. doi: 10.3390/ijms26136166 40649944 PMC12250127

[55] Galarza Fortuna GM Peres L Nazarenko E De Menezes Silva Corraes A Hovanky VN Shune L . Safety and efficacy of BCMA-directed chimeric antigen receptor T-cell therapy for the treatment of plasma cell leukemia. Blood Adv. (2025) 9:6009–18. doi: 10.1182/bloodadvances.2025016966 40875887 PMC12686786

[56] Tan M Cook J Gonsalves WI Zanwar S Kapoor P Abdallah N . CAR T-cell therapy outcomes in plasma cell leukemia. Blood Immunol Cell Ther. (2026) 2:100033. doi: 10.1016/j.bict.2026.10003342574925

[57] Gaballa M Julian K Afrough A Hansen D Goel U De Menezes Silva Corraes A . Real world outcomes of bispecific T-cell engagers in plasma cell leukemia. Blood. (2025) 146:823. doi: 10.1182/blood-2025-823

[58] Yan W Du C Zou H Zhang S Deng S Qiu L . BCMA CAR T-cell therapy in newly diagnosed primary plasma cell leukemia ineligible for transplantation: An open label, single-arm, phase 2 study (CAREMM-002). Blood. (2025) 146:257. doi: 10.1182/blood-2025-257

[59] Xu J Bai Q Liu Y Yan W Zhang S Huang W . CART–ASCT–CART2 sandwich regimen as frontline therapy: A phase II trial in patients with primary plasma cell leukemia. Blood. (2025) 146:4067. doi: 10.1016/s2152-2650(25)03570-031792650

[60] Mehta S Rizvi SS Rodriguez AMA . The evolving landscape of chimeric antigen receptor (CAR) T-cell therapy for plasma cell leukemia: A systematic review of early outcomes. J Clin Oncol. (2026) 44:e19508. doi: 10.1200/jco.2026.44.16_suppl.e1950842697810

[61] Afrough A Dima D Razzo B Goel U Sannareddy A Pasvolsky O . The impact of extramedullary and paraskeletal plasmacytomas on treatment outcomes in multiple myeloma treated with teclistamab: U.S. Myeloma Immunotherapy Consortium real-world experience. Blood Cancer J. (2025) 16:12. doi: 10.1038/s41408-025-01414-6 41315206 PMC12789570

[62] Dima D Afrough A Goel U Grajales-Cruz AF Khouri J Julian K . Teclistamab for patients with heavily pretreated relapsed/refractory multiple myeloma and renal impairment. Blood Adv. (2025) 9:3408–17. doi: 10.1182/bloodadvances.2025016059 40198766 PMC12274829

[63] Tan CR Asoori S Huang C-Y Brunaldi L Popat R Kastritis E . Real-world evaluation of teclistamab for the treatment of relapsed/refractory multiple myeloma (RRMM): an International Myeloma Working Group Study. Blood Cancer J. (2025) 15:53. doi: 10.1038/s41408-025-01259-z 40175336 PMC11965530

[64] Costa LJ Banerjee R Mian H Weisel K Bal S Derman BA . International myeloma working group immunotherapy committee recommendation on sequencing immunotherapy for treatment of multiple myeloma. Leukemia. (2025) 39:543–54. doi: 10.1038/s41375-024-02482-6 39870767 PMC11879857

[65] van de Donk N Moreau P San-Miguel JF Mateos MV Dimopoulos MA Zweegman S . Optimising T-cell immunotherapy in patients with multiple myeloma: practical considerations from the European Myeloma Network. Lancet Haematol. (2025) 12:e635–49. doi: 10.1016/s2352-3026(25)00117-6 40580975

[66] Mina R Mylin AK Yokoyama H Magen H Alsdorf W Minnema MC . Patient-reported outcomes following ciltacabtagene autoleucel or standard of care in patients with lenalidomide-refractory multiple myeloma (CARTITUDE-4): results from a randomised, open-label, phase 3 trial. Lancet Haematol. (2025) 12:e45–56. doi: 10.1016/s2352-3026(24)00320-x 39756844 PMC12321245

[67] Delforge M Patel K Eliason L Dhanda D Shi L Guo S . Health-related quality of life in patients with triple-class exposed relapsed and refractory multiple myeloma treated with idecabtagene vicleucel or standard regimens: patient-reported outcomes from the phase 3, randomised, open-label KarMMa-3 clinical trial. Lancet Haematol. (2024) 11:e216–27. doi: 10.1016/s2352-3026(24)00005-x 38423700

[68] Schinke C Touzeau C Oriol A Mateos MV Stevens D Rasche L . Talquetamab improves patient-reported symptoms and health-related quality of life in relapsed or refractory multiple myeloma: Results from the phase 1/2 MonumenTAL-1 study. Cancer. (2025) 131:e35927. doi: 10.1002/cncr.35927 40631904 PMC12239699

[69] Yamamoto C Minakata D Yokoyama D Furuki S Noguchi A Koyama S . Cost-effectiveness of anti-BCMA chimeric antigen receptor T cell therapy in relapsed/refractory multiple myeloma. Transplant Cell Ther. (2024) 30:118.e1–e15. doi: 10.1016/j.jtct.2023.10.001 37802181

[70] Hansen DK Lu X Puglianini OC Sorensen S Usmani SZ Zhang E . Cost-per-responder analysis of patients with lenalidomide-refractory multiple myeloma receiving ciltacabtagene autoleucel in CARTITUDE-4. Front Immunol. (2024) 15:1408892. doi: 10.3389/fimmu.2024.1408892 39234256 PMC11372240

[71] Samur MK Fulciniti M Aktas Samur A Bazarbachi AH Tai Y-T Prabhala R . Biallelic loss of BCMA as a resistance mechanism to CAR T cell therapy in a patient with multiple myeloma. Nat Commun. (2021) 12:868. doi: 10.1038/s41467-021-21177-5 33558511 PMC7870932

[72] Lee H Neri P Bahlis NJ . BCMA- or GPRC5D-targeting bispecific antibodies in multiple myeloma: efficacy, safety, and resistance mechanisms. Blood. (2024) 143:1211–7. doi: 10.1182/blood.2023022499 38194680

[73] Hosoya H Carleton M Tanaka K Sworder B Syal S Sahaf B . Deciphering response dynamics and treatment resistance from circulating tumor DNA after CAR T-cells in multiple myeloma. Nat Commun. (2025) 16:1824. doi: 10.1038/s41467-025-56486-6 39979252 PMC11842827

[74] Munawar U Thurner J Nerreter S Nerreter T Leipold AM Han S . Loss of GPRC5D enhances the proliferative capacity and competitive fitness of myeloma upon anti-GPRC5D immunotherapy. Leukemia. (2026) 40:1227–39. doi: 10.1038/s41375-026-02920-7 41917280 PMC13233306

[75] Chen H Yu T Lin L Xing L Cho SF Wen K . γ-secretase inhibitors augment efficacy of BCMA-targeting bispecific antibodies against multiple myeloma cells without impairing T-cell activation and differentiation. Blood Cancer J. (2022) 12:118. doi: 10.1038/s41408-022-00716-3 35973981 PMC9381512

[76] Friedrich MJ Neri P Kehl N Michel J Steiger S Kilian M . The pre-existing T cell landscape determines the response to bispecific T cell engagers in multiple myeloma patients. Cancer Cell. (2023) 41:711–725.e6. doi: 10.1016/j.ccell.2023.02.008 36898378

[77] Fang J Zhou F . BCMA-targeting chimeric antigen receptor T cell therapy for relapsed and/or refractory multiple myeloma. Ann Hematol. (2024) 103:1069–83. doi: 10.1007/s00277-023-05444-7 37704875

[78] Kumar S Mateos MV Ye JC Atrash S Magen H Quach H . Dual targeting of extramedullary myeloma with talquetamab and teclistamab. N Engl J Med. (2026) 394:51–61. doi: 10.1056/nejmoa2514752 41358582

[79] Chari A van de Donk N Dholaria B Weisel K Mateos MV Goldschmidt H . Talquetamab plus daratumumab for the treatment of relapsed or refractory multiple myeloma in the TRIMM-2 study. Blood. (2025) 146:2902–13. doi: 10.1182/blood.2025029360 40983036

[80] Jurgens EM Firestone RS Chaudhari J Hosszu K Devlin SM Shah UA . Phase I trial of MCARH109, a G protein-coupled receptor class C group 5 member D (GPRC5D)-targeted chimeric antigen receptor T-cell therapy for multiple myeloma: An updated analysis. J Clin Oncol. (2025) 43:498–504. doi: 10.1200/jco-24-01785 39631041 PMC11798713

[81] Mailankody S Devlin SM Landa J Nath K Diamonte C Carstens EJ . GPRC5D-targeted CAR T cells for myeloma. N Engl J Med. (2022) 387:1196–206. doi: 10.1056/nejmoa2209900 36170501 PMC10309537

[82] Pan M Wang D Xu J Jin S Wang Y Tao Y . The fully human anti-GPRC5D CAR T-cell therapy RD118 induces durable remissions in relapsed/refractory multiple myeloma. Blood. (2026) 147:513–9. doi: 10.1182/blood.2025030559 41118600

[83] Bal S Htut M Nadeem O Anderson LD Gregory T Kocoglu M . Arlocabtagene autoleucel-a GPRC5D-targeted CAR T-cell therapy in heavily pretreated relapsed/refractory multiple myeloma. Blood. (2026) 148(10):1240–50. doi: 10.1182/blood.2025030750 42233419 PMC13598863

[84] Pillarisetti K Yang D Luistro L Yao J Smith M Vulfson P . Ramantamig (JNJ-79635322), a novel T-cell-engaging trispecific antibody targeting BCMA, GPRC5D, and CD3, in multiple myeloma models. Blood. (2026) 147:834–47. doi: 10.1182/blood.2025030027 41100731 PMC13000934

[85] Li J Li Z Li C He P Luo J Fu R . Initial results of the first-in-human phase 1 study of IBI3003, a novel trispecific antibody targeting GPRC5D, BCMA and CD3, in patients with relapsed or refractory multiple myeloma. Blood. (2025) 146:702. doi: 10.1182/blood-2025-702

[86] Lichtman EI Khot A Augustson B Levitz D Sia H Doo NW . Phase 1, first-in-human study of ISB 2001: A BCMAxCD38xCD3-targeting trispecific antibody for patients with relapsed/refractory multiple myeloma (RRMM)—Dose escalation (DE) results. J Clin Oncol. (2025) 43:7514. doi: 10.1200/jco.2025.43.16_suppl.751442697810

[87] Richter J Thomas SK Krishnan AY Laubach JP Cohen AD Trudel S . Cevostamab in patients with heavily pretreated relapsed/refractory multiple myeloma (RRMM): Updated results from an ongoing phase I study demonstrate clinically meaningful activity and manageable safety and inform the doses and regimen for combination studies. Blood. (2024) 144:1021. doi: 10.1182/blood-2024-199542

[88] Sam I Ben Hamouda N Alkatrib M Gonnin C Siska PJ Oudard S . The CD70-CD27 axis in cancer immunotherapy: predictive biomarker and therapeutic target. Clin Cancer Res. (2025) 31:2872–81. doi: 10.1158/1078-0432.ccr-24-2668 40388162

[89] McEarchern JA Smith LM McDonagh CF Klussman K Gordon KA Morris-Tilden CA . Preclinical characterization of SGN-70, a humanized antibody directed against CD70. Clin Cancer Res. (2008) 14:7763–72. doi: 10.1158/1078-0432.ccr-08-0493 19047103

[90] Kasap C Izgutdina A Patiño-Escobar B Kang AS Chilakapati N Akagi N . Targeting high-risk multiple myeloma genotypes with optimized anti-CD70 CAR T cells. Blood. (2025) 146:819–33. doi: 10.1182/blood.2024025536 40359480 PMC12783522

